# Murphy at Moscow

**Published:** 1897-11

**Authors:** 


MURPHY AT MOSCOW.—Dr. Murphy, of "Button" fame, astonished the natives at the International Medical Congress in Russia. "Papers read in English," says the *New York Medical Record*, "usually receive the most brutal treatment; not so with that of Dr Murphy; it was listened to with marked attention, and his instruments were examined with great interest." Murphy is a very young man, but is world-wide famous. In this instance he reported cutting down on the right subclavian artery which had been cut by a bullet, and a large hæmatoma had formed. He cut out five-eighths of an inch of the artery, stitched the two ends together, and got pulse at the wrist immediately. A surgeon who can do this, American tho' he be, is entitled to be listened to.

---

DO NOT LET UP on the demand for a Public Health Department, with a commissioner in the Cabinet, It is an absolute essential if ever we expect to see the conclusions of science, as to sanitary matters, crystalized into laws. One reason of our bungling and inconsistent laws is, they are made by men who know nothing of sanitation; don't want to be told, and could not understand if told, and who are notoriously averse to being advised. Write to your congressmen and senators, urging their support of such a measure.

